# Economic and environmental impact assessment of sustainable future irrigation practices in the Indus Basin of Pakistan

**DOI:** 10.1038/s41598-021-02913-9

**Published:** 2021-12-06

**Authors:** Muhammad Muzammil, Azlan Zahid, Lutz Breuer

**Affiliations:** 1 Institute for Landscape Ecology and Resources Management (ILR), Research Centre for BioSystems, Land Use and Nutrition (IFZ), Justus Liebig University Giessen, 35392 Giessen, Germany; 2 Department of Irrigation and Drainage, University of Agriculture, Faisalabad, 38040 Pakistan; 3 Texas A&M AgriLife Research, Texas A&M University System, Dallas, TX 75252 USA; 4 Centre for International Development and Environmental Research (ZEU), Justus Liebig University Giessen, 35390 Giessen, Germany

**Keywords:** Environmental sciences, Hydrology

## Abstract

Pakistan’s agriculture is characterized by insecure water supply and poor irrigation practices. We investigate the economic and environmental feasibility of alternative improved irrigation technologies (IIT) by estimating the site-specific irrigation costs, groundwater anomalies, and CO_2_ emissions. IIT consider different energy sources including solar power in combination with changes in the irrigation method. The status quo irrigation costs are estimated to 1301 million US$ year^−1^, its groundwater depletion to 6.3 mm year^−1^ and CO_2_ emissions to 4.12 million t year^−1^, of which 96% originate from energy consumption and 4% via bicarbonate extraction from groundwater. Irrigation costs of IIT increase with all energy sources compared to the status quo, which is mainly based on diesel engine. This is because of additional variable and fixed costs for system’s operation. Of these, subsidized electricity induces lowest costs for farmers with 63% extra costs followed by solar energy with 77%. However, groundwater depletion can even be reversed with 35% rise in groundwater levels via IIT. Solar powered irrigation can break down CO_2_ emissions by 81% whilst other energy sources boost emissions by up to 410%. Results suggest that there is an extremely opposing development between economic and ecological preferences, requiring stakeholders to negotiate viable trade-offs.

## Introduction

Agriculture of Pakistan is based on irrigation where rainfall marginally meets 15% of crop water requirements^1^. Irrigated agriculture is associated with the Indus Basin, which provides irrigation water on a supply basis. Surface water resources are unable to fulfill the actual irrigation demands owing to the high evapotranspiration and salinity environment in the plain^2^. Groundwater covers 40–60% of the irrigation needs to meet the deficit in surface water supplies^3^. The intensive groundwater use results in a decline of the water table and accumulation of soil salinity, which originates from saline pockets of the aquifers^4^. Meanwhile, the increasing trend of groundwater pumping has become energy exhaustive. It reduces the farmer’s income because of high irrigation costs and leads to massive greenhouse gas releases, mainly CO_2_ emissions, through energy consumption^5^. Furthermore, the role of groundwater depletion in greenhouse emissions is still unaccounted, which can be a significant emission source associated with bicarbonate extraction^6,7^. Despite the poor situation of water resources availability in the country, inefficient irrigation methods dominate in the region, with losses of up to 50% of available water in the fields^8^.

In many arid and semi-arid countries, where water resources are limited and depleting rapidly, there is pressure to reduce water consumption for the water security of the growing population^9^. Various management approaches have been suggested to save water, including deficit irrigation, soil mulching, conservation tillage, cultivation of drought resistance or low water demanding crops^10^. Previous studies indicate that improved irrigation technologies (IIT) enable farmers to cope with water scarcity and insecure water supply^10–12^. However, the impact of IIT remains a critical topic for sustainable irrigation. The consequences of IIT vary among regions because of differences in cost-benefits, off-farm environmental impacts, and social preferences^13^. Economic impact assessment should be part of the evaluation process and support the decision-making. It can be used to project the levels of economic activity generated in a region by a specific project or alternatively without that project^14^. For example, Zou et al.^15^ analyzed water-saving strategies based on the climate change response for China and proposed that channel lining is a preferable strategy from an economic perspective compared to pressurized irrigation practices. Mahinda et al.^16^ investigated the economic impact of sorghum production via drip irrigation in semi-arid regions of Tanzania and recommended that two irrigations per day are beneficial to get higher economic returns. Narayanamoorthy et al.^17^ studied the economic impact of drip irrigation on vegetable crops and their findings indicate that the pressurized irrigation system offers high net returns compared to conventional irrigation methods.

However, irrigation development can also have severe environmental effects at regional and basin levels^18–20^. For example, Panday et al.^21^ studied the environmental impact of canal irrigation in India and concluded that construction of canal is beneficial to enhance the crop production, but it resulted in waterlogging and rising salinity. Daccache et al.^22^ projected that a pressurized irrigation system is capable to increase irrigation efficiency, but CO_2_ emissions increase due to additional energy consumption compared to a gravity-fed surface irrigation system. Shekhar et al.^23^ showed that technology changes could have the potential to mitigate groundwater depletion through pressure reduction on water resources. However, the lower percolation from fields with improved water saving irrigation techniques may reduce aquifer recharge^24^. Mojid et al.^25^ revealed that high-efficiency irrigation technologies reduce agriculture water consumption, but large-scale adoption can lead to negative impacts on groundwater dynamics and the regional water cycle because of lower percolation rates to recharge the groundwater. Farsi Aliabadi et al.^26^ investigated the environmental impacts of IIT supported by subsidized energy supply in Iran and found that such programs are not likely to overcome groundwater depletion. In Pakistan, the potential of IIT related to water saving have been recognized. Several studies revealed that it is possible to overcome water scarcity in Pakistan through the adoption of high-efficiency irrigation systems^8,27,28^. Meanwhile, previous studies show that future power supply for IIT should consider changes in the energy source, including solar power supply^29,30^. Nevertheless, the economic and environmental impacts of these technologies are still unknown over the status-quo irrigation settings. An inclusive analysis of the cost-effectiveness of IIT coping with ecological impact can support economic development and environmental sustainability in the region.

In this study, we compare the economic and environmental impacts of the status-quo irrigation settings with alternative IIT. We use a coupled economic-environmental-modeling framework to estimate the irrigation costs, groundwater depletion, and CO_2_ emissions to understand the return on investment and environmental effects. We consider improved, more sustainable irrigation technologies that differ from the status-quo irrigation practices in terms of water consumption, irrigation costs, and energy use. As the water consumption via IIT is lower than that of conventional irrigation, the effect of groundwater recharge through surplus irrigation is diminishing, which we take also into account. Furthermore, improving the established irrigation system needs a high initial investment and, in the case where the gravity-fed irrigation system is replaced, additional operational energy costs and associated CO_2_ emissions come into play, which are also analyzed.

The objectives of the current study are: (1) to investigate the economic impact of IIT over status-quo irrigation practices, (2) to compare groundwater depletion and CO_2_ emissions of the status-quo irrigation settings with improved irrigation practices, and (3) to develop alternative scenarios for IIT and identify sustainable energy use options in the irrigation agriculture of Pakistan.

### Description of the study area

**Figure 1:**
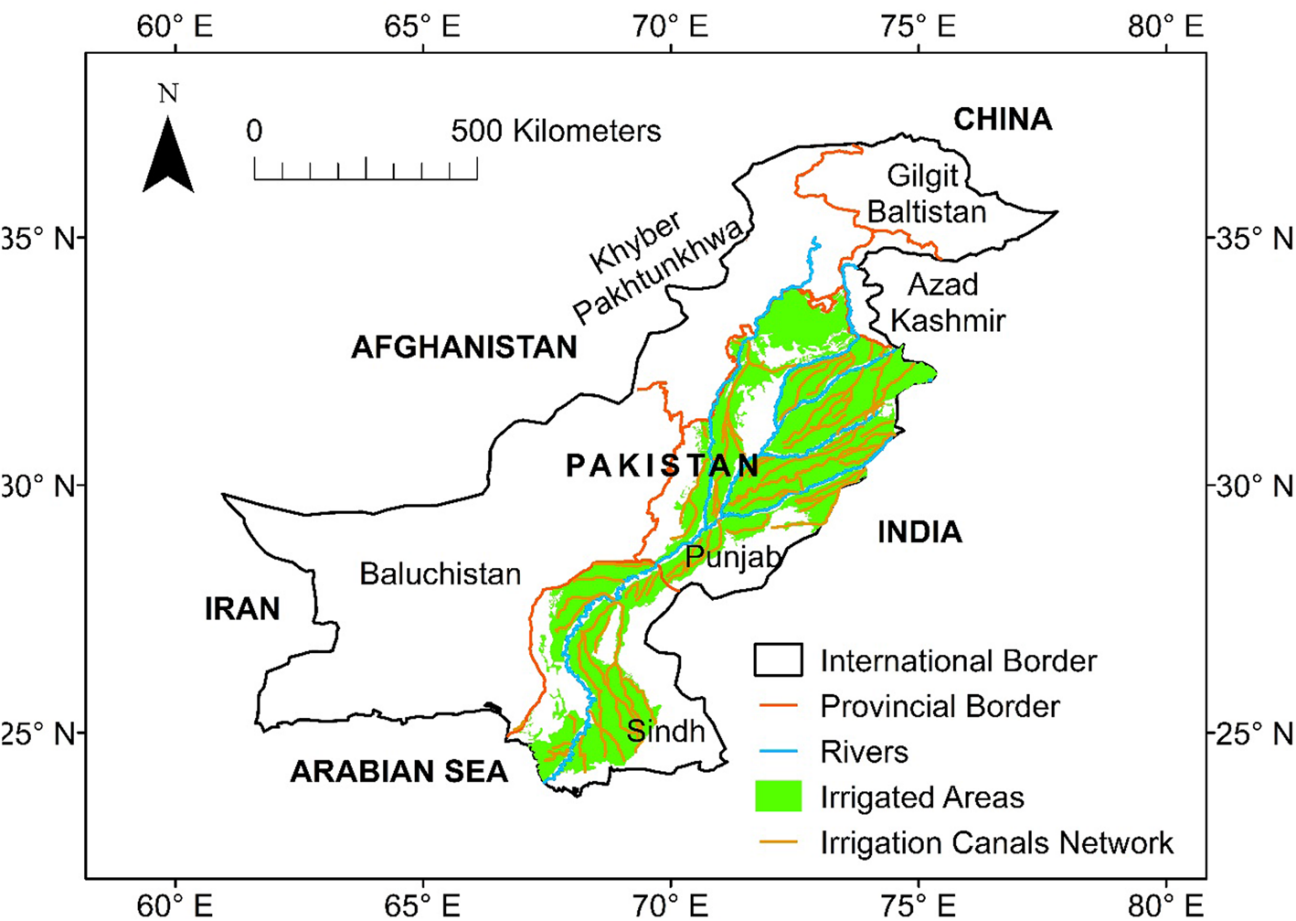
Map of the study area. The figure is generated in ArcGIS version 10.6.1 (https://www.esri.com/en-us/arcgis/products/index).

The study focuses on the irrigated areas of Punjab and Sindh provinces in the Indus basin of Pakistan. Together, these cover 17 million ha (Fig. 1), representing 90% of the total irrigated area in the country. The topography of the plain falls from north to south, ranging from 540 to 4 m above mean sea level. The basin has an arid to semi-arid climate with complex hydrological processes due to spatial and temporal variation in the rainfall, temperature, land use, and water consumption. The average annual rainfall amounts to 379 mm (2002–2018), while maximum temperature ranges from 34 to 44 °C in the summer (Apr–Sep) and 20–28 °C in the winter (Dec–Feb). The annual potential evapotranspiration varies from 1200 to 2050 mm from the north to the south. Crops are harvested in two cropping seasons called Kharif (wet season; Apr–Sep) and Rabi (dry season; Oct–Mar). Sugarcane, cotton, and rice are dominant crops in the Kharif while wheat is a major crop in the Rabi season.

There are five major tributaries to the Indus (Indus, Chenab, Ravi, Jhelum, and Sutlej), which supply irrigation water via a network of canals and watercourses. The provincial governments distribute the surface water among farmers according to the landholding size and collect the water charges two times in a year in the Kharif and Rabi seasons. The water charges vary from province to province i.e., the Punjab government collects at a flat rate despite which crop is grown while it varies in Sindh by crop to crop. Farmers use additional groundwater recourses via private units (tubewells), operate with diesel engines or mains power for groundwater pumping. The government provides subsidized electricity to farmers. However, diesel operated tubewells are common among farmers with 87% of share because they have a lower initial investment than electric operated tubewells. Crops are widely irrigated via surface irrigation with an application efficiency of 45–60%. Improved irrigation systems (drip and sprinkler) are installed only in a limited area (50,000 ha) through a subsidized program of the World Bank and the government of Punjab in the frame of the Punjab Irrigated-Agriculture Productivity Improvement Project (PIPIP).

## Results

### Water consumption and irrigation costs

**Figure 2:**
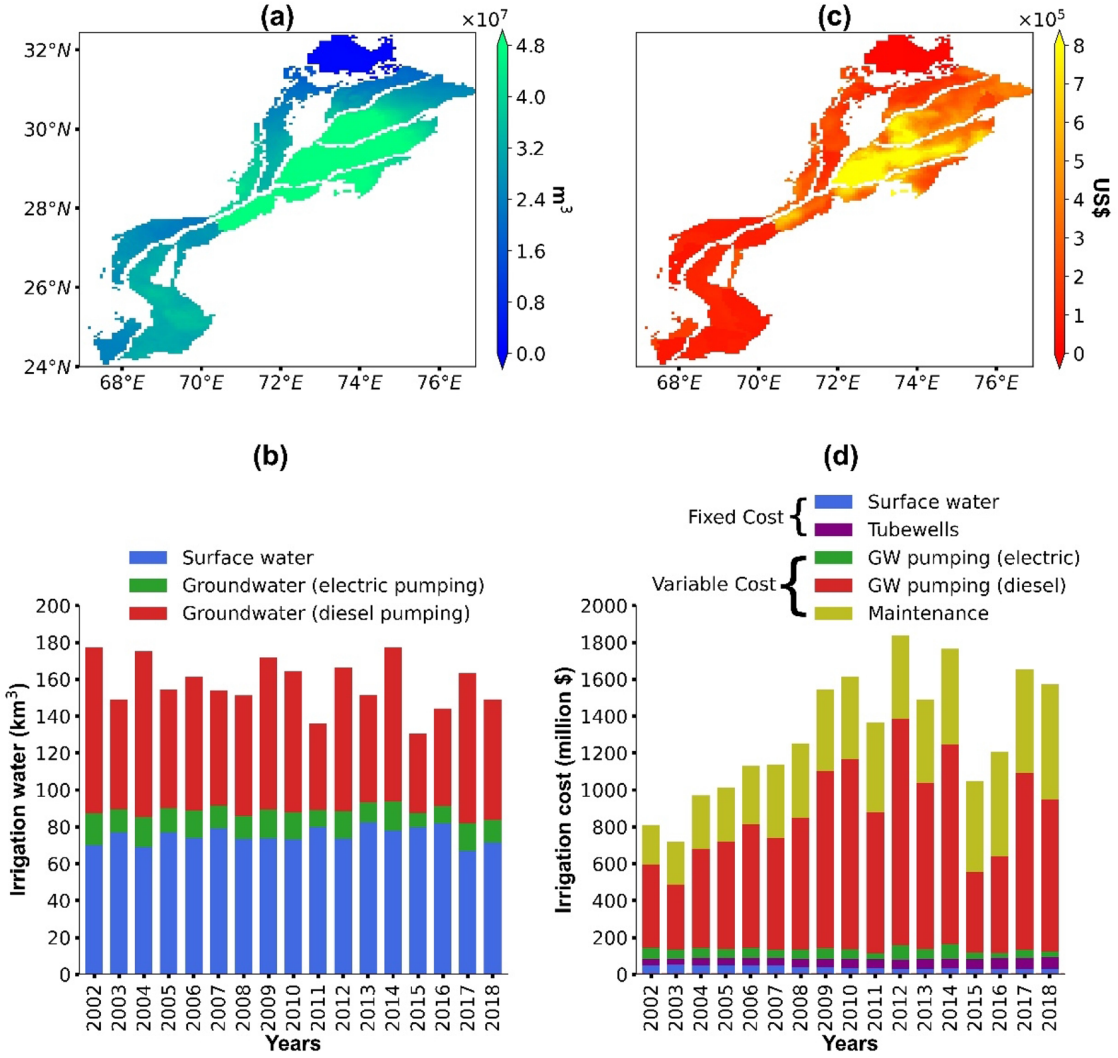
Irrigation water consumption (**a**, **b**) and irrigation cost (**c**, **d**) from 2002 to 2018.

The shares of surface and groundwater in irrigation water are shown in Fig. S1 as a supplementary material. The irrigation water consumption (IRR_area_) and the total irrigation costs (TC_area_) for 2002–2018 are presented in Fig. 2. Results show that the southern part of Punjab has the highest IRR_area_ while the upper portion of Punjab and the whole parts of Sindh have relatively lower IRR_area_ (Fig. 2a). We find strong inter-annual variation in IRR_area_ with the highest in 2002 (177 km^3^ year^−1^) and the lowest in 2015 (130 km^3^ year^−1^) (Fig. 2b). A Mann–Kendall test reveals that there is no trend in IRR_area_ from 2002 to 2018 (p = 0.23). Average IRR_area_ is estimated to 157 km^3^ year^−1^, of which groundwater accounts for 52% (82 km^3^ year^−1^) and surface water contributes to 48% (75 km^3^ year^−1^). Diesel pumping has the largest share in groundwater abstraction with 83%, followed by electric pumping of 17%. Results of TC_area_ also show a substantial variation in space and year-to-year (Fig. 2c,d). The southern region of Punjab has the highest TC_area_ compared to other parts of the study area (Fig. 2c). The highest TC_area_ are calculated for 2014 (1,837 million US$) and the lowest one for 2003 (718 million US$) (Fig. 2d). We find an overall significant increasing trend for TC_area_ from 2002 to 2018 (R^2^ = 0.43, slope = 41.6, p = 0.001). The years 2015 and 2016 are striking with lower costs, which are due to the combined effect of a lower IRR_area_ and reduced fuel prices compared to other years. The average TC_area_ for 2002–2018 are calculated to 1,301 million US$, of which fixed cost (TFC_area_) components account for 8% (85 million US$) and variable costs (TVC_area_) account for by far the largest amount (1216 million US$). Groundwater pumping costs (GPC_area_) have the largest share in TC_area_ with 60%, followed by maintenance costs (MC_area_; 32%), surface water prices (SWP_area_; 3%), and tubewell construction costs (TCC_area_; 5%). Diesel pumping costs (GPC_area(d)_) have a dominant part in GPC_area_ with 93%, while the electric pumping cost (GPC_area(e)_) holds only 7%.

### Estimates of groundwater depletion

**Figure 3:**
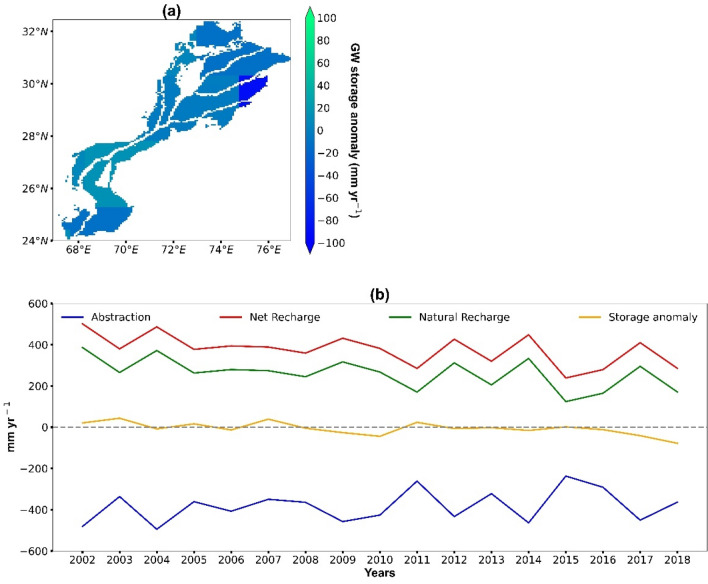
(**a**) Average groundwater depletion from 2002 to 2018, (**b**) Time series trend (2002–2018) of groundwater abstraction, net recharge, natural recharge, and storage anomaly.

We project the groundwater storage from 2002 to 2018 by estimating the groundwater recharge and abstraction in the study area. The results show that the northern part of the plain (Punjab province) faces the largest depletion rate (− 11 mm year^−1^) while an increase in groundwater level (4 mm year^−1^) is observed in the southern part of the plain (Sindh province) (Fig. 3a). Overall, the groundwater storage anomaly is significantly decreasing (R^2^ = 0.39, slope =  − 3.93, p = 0.02) in the study area from 2002 to 2018 (Fig. 3b) at a rate of − 6.3 mm year^−1^ (− 1.35 km^3^ year^−1^). Annual differences are substantial, with the highest depletion rate in the year 2018 (− 78 mm year^−1^; − 16.7 km^3^ year^−1^) and the largest surplus in groundwater storage in 2003 (43 mm year^−1^; 9.2 km^3^ year^−1^). Overall, we do neither find significant trends for net groundwater recharge (p = 0.06) nor for abstraction (p = 0.38). Further, the average net recharge rate is estimated to 380 mm year^−1^, of which 69% (263 mm year^−1^) are contributed by from natural resources (precipitation, and particular leaching from rivers, water bodies and canals) while the cropping fields add another 31% (117 mm year^−1^) as percolation losses from unproductive irrigation.

### Estimates of CO_2_ emissions

**Figure 4:**
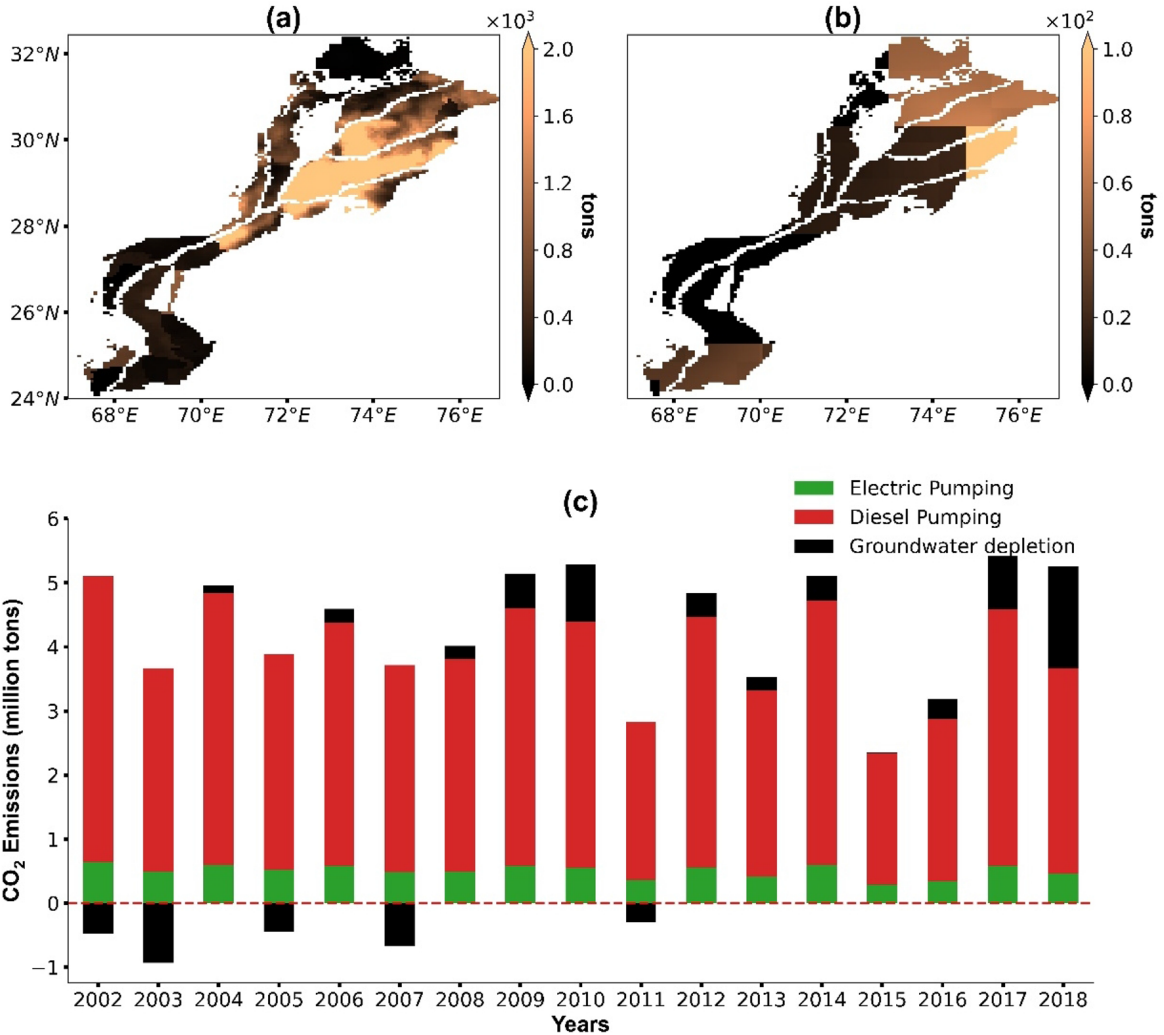
Average annual CO_2_ emissions from (**a**) energy consumption and (**b**) groundwater depletion, (**c**) temporal development of CO_2_ emissions from 2002 to 2018. [Note that the color-codes in the maps (**a**) and (**b**) vary by a factor of 10].

We estimate CO_2_ emissions from 2002 to 2018 according to the emission sources, i.e., energy consumption and bicarbonate extraction from depleted groundwater volume (Fig. 4). The southern part of Punjab depicts the highest CO_2_ emissions from energy consumption (Fig. 4a) while the upper portion of Punjab shows the highest CO_2_ emissions due to groundwater depletion (Fig. 4b). The results further reveal that about 4.12 million t CO_2_ year^−1^ are emitted in the plain, of which 96% (3.95 million t year^−1^) result from energy consumption while 4% (0.17 million t year^−1^) are stemming from groundwater depletion. The largest CO_2_ emissions are produced in the year 2018 (5.42 million t) and the lowest one in 2015 (2.15 million t) (Fig. 4c). Further, CO_2_ emissions from groundwater depletion are highly variable over time with a maximum in 2018 (1.58 million t). For several years, we found even negative values (i.e., an increase of the CO_2_ storage) due to a surplus of groundwater recharge over groundwater abstraction. This results in rather substantial net storage of CO_2_ in 2003 (− 0.93 million t). With regard to the energy source, diesel pumping has a larger share (87%) than CO_2_ emissions from electric pumping.

### Scenario analysis

**Figure 5:**
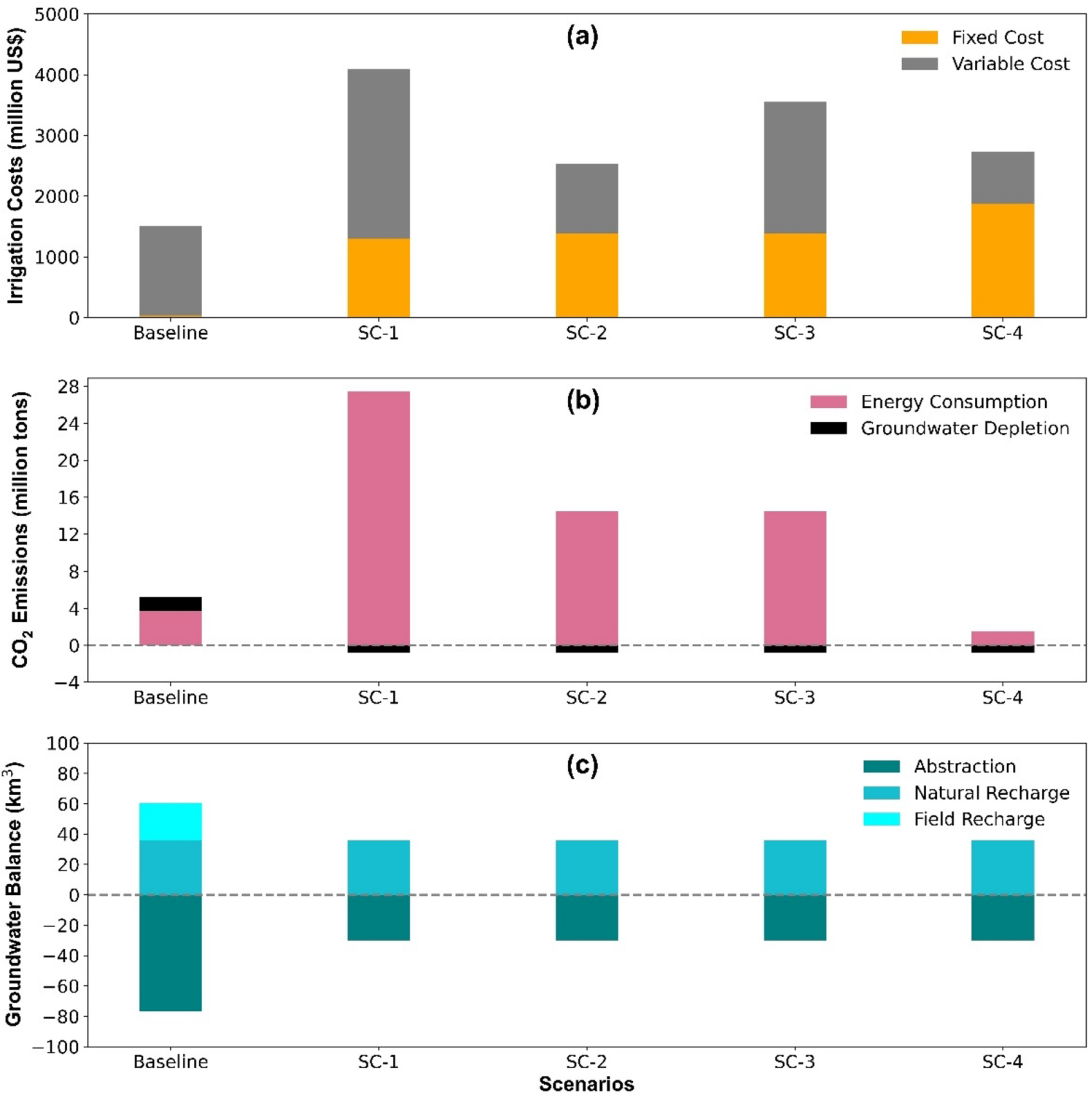
Scenarios analysis for (**a**) irrigation costs (**b**) CO_2_ emissions and (**c**) groundwater balance, via IIT.

Scenarios are investigated to derive the optimum energy source for IIT and compare the results with the status-quo irrigation method. We establish four scenarios SC1-4 to identify the effect of IIT on TC_area_, groundwater depletion, and CO_2_ emissions for more sustainable irrigation practices by using different energy sources in each scenario. The changes in TC_area_, groundwater depletion, and CO_2_ emissions for all scenarios are presented in Fig. 5 and Table S2 as a supplementary material. In SC-1, we change the gravity driven status-quo irrigation settings with IIT and consider diesel as the primary energy source. The results indicate that TC_area_ and CO_2_ emissions increase up to 170% and 410%, respectively, while the groundwater depletion is reduced by up to 135%. SC-2 focuses on changing the status quo irrigation settings with IIT that run on subsidized electricity from mains power. We find an increase in TC_area_ and CO_2_ emissions of up to 63% and 165%, respectively. Meanwhile, the groundwater depletion rate decreases by up to 135%. The scenario SC-3 has the same settings as SC-2 but we use actual prices for electricity. In consequence, we observe an increase in TC_area_ of up to 130% of the baseline scenario. In SC-4, solar-powered IIT are used instead of the surface irrigation method. The results show that TC_area_ increase by up to 77% while CO_2_ emissions and groundwater depletion are reduced by up to 81% and 135%, respectively.

## Discussions

### Economic impact of irrigation methods

In the status-quo conditions, the average IRR_area_ in the study area is 157 km^3^ year^−1^, of which surface water contributes 48% and groundwater 52%. Despite the small difference in water consumption from surface water and groundwater, there is a vast margin between prices with 3% for surface water and 63% for groundwater of TC_area_ (1301 million US$), respectively. Alternatively, scenarios indicate that IIT can reduce IRR_area_ by 32%, which could lead to a reduction in groundwater share of up to 61%, with at the same time 55% decreasing GPC_area_. However, IIT raise TC_area_ owing to the initial and running costs of the system. Scenarios specify that the operation of IIT via subsidized electricity is an optimal scenario among others for farmer’s perspective where TC_area_ increase by 63% compared to the status quo. Solar energy is the second most feasible power source when no subsidized electricity is at hand, but still, TC_area_ increase by 77% compared to that of the status quo. Highest costs are found for diesel operated systems which boost TC_area_ by up to 170%. In short, the economic benefits of IIT are insufficient over the status-quo practices to cover the additional expenditure of the irrigation system. This is in line with various other studies that recognized that IIT can increase farmer’s expenditures via capital investments and running costs^31–33^. For example, Paramar et al.^34^ examined the barriers faced by farmers in India in adopting drip irrigation and found that the high initial cost is a major economic constraint to adoption of the technology. Rodrigues et al.^35^ studied the comparative advantages of drip and sprinkler irrigation in southern Brazil and concluded that economic benefits from water-saving technologies are insufficient to recover the initial costs of the system. Numerous studies revealed that the implication of IIT is a challenge owing to an extra burden of investment compared to surface irrigation. In Pakistan, despite the various awareness campaigns in the last three decades to introduce IIT, farmers are still not willing to adopt the technologies because of the high initial costs of the system. Thus, governments should provide subsidies to farmers for sustainable water consumption^36–38^, such as in the World Bank funded Punjab Irrigated-Agriculture Productivity Improvement Project with a size of 50,000 ha. Such types of projects have the capability to promote water-saving technologies among farmers. However, it is doubtful that such a technical shift is sustainable from an economic viewpoint.

Part of this problem might be arising from the very low surface water prices in Pakistan, which do not promote changing towards more efficient, but costly irrigation technologies. Qamar et al.^39^ studied the implication strategies of IIT in the Indus basin of Pakistan and concluded that the surface water prices should be higher to promote IIT among farmers. We recommend that a comprehensive analysis should be conducted to study the adoption strategies of IIT by changing the water prices. Such an analysis should not only consider pure economic aspect, but also take into account societal barriers and personal preferences as well as choices from farmers.

### CO_2_ emissions from irrigation practices

We estimated CO_2_ emissions from irrigation practices in the Indus basin of Pakistan by assuming emissions from energy consumption and bicarbonate extraction. At the status-quo settings, diesel or electric pumps are used to pump groundwater, which produces 96% of the total CO_2_ emissions (3.95 million t). Our estimates indicate that bicarbonate extraction is not a significant emissions source, amounting to about 4% of the total CO_2_ emissions (0.17 million t), although groundwater makes up a significant part of the irrigation water in the Indus basin. Mishra et al.^6^ estimated the annual CO_2_ emissions from groundwater bicarbonate extraction to around 0.72 million t, which is not a significant emissions source either compared to energy consumption through groundwater pumping. Wood and Hyndman^7^ calculated CO_2_ emissions from bicarbonates extraction in the USA and determined that annual 1.7 million t of CO_2_ are released from this source. Despite a tenfold higher rate as compared to the groundwater mediated CO_2_ emissions in the Indus basin, the total share of bicarbonate extraction on US CO_2_ emissions is small with less than 0.5% (estimated from data published by Wood and Hyndman^7^).

Past studies proposed several strategies to reduce CO_2_ emissions from groundwater pumping. For example, Shah and Kishore^40^ recommended on-site solar and wind energy for groundwater pumping. However, the authors show serious concern that the availability of renewable energy will encourage the farmers to pump additional groundwater because of the currently low pumping costs. Dhillon et al.^41^ projected that an improvement in pumping plant efficiency could also reduce CO_2_ emissions. Zou et al.^42^ showed indirect effects through general water savings of improved irrigation systems and subsequent lower CO_2_ emissions because of a reduced groundwater demand. However, IIT might require further energy to run the system, which in turn can increase overall CO_2_ emissions. Daccache et al.^22^ studied the environmental impact of irrigation practices in the Mediterranean region of Spain. Similar to our results, they revealed that CO_2_ emissions increased by 135% for IIT compared to the old-fashioned, gravity-based surface irrigation method.

We estimate CO_2_ emissions for different scenarios of IIT by combining emissions from groundwater pumping and irrigation system operation. Our results indicate that diesel engines and mains power electricity are both detrimental energy sources for advancing irrigation technologies compared to the status-quo settings, simply because of the huge increase in CO_2_ emissions by 410% and 165%, respectively. However, solar energy operating systems are most effective, which can reduce CO_2_ emissions even of the status-quo technology by 81%. Many studies revealed that solar energy is the best option for IIT for sustainable development in a region or basin^43–45^.

### Groundwater depletion

In the study area, the average groundwater depletion is 6.3 mm year^−1^, which is comparatively low. For example, Long et al.^46^ estimated the groundwater depletion to 31 mm year^−1^ in the Northwest Indian Aquifer. Shen et al.^47^ investigated groundwater storage anomaly in the Hai River Basin China and reported that groundwater is depleting at a rate of 17 mm year^−1^. Dangar et al.^48^ showed that groundwater depleted significantly during the period of 2002–2016 in the Ganga river basin India with a rate of 15 mm year^−1^. Voss et al.^49^ assessed the groundwater storage anomaly in the Tigris-Euphrates region of Iran by using GRACE data and projected that groundwater level drops at a rate of 17 mm year^−1^.Despite the lower depletion rate in the Indus basin, our estimates show that the groundwater depletion trend is increasing from 2002 to 2018. Tang et al.^50^ confirmed that groundwater storage is diminishing in the Indus basin. It has been predicted that the depletion rate in the Indus basin will increase by 50% in 2050 compared to the groundwater depletion trend in 2005^51^. We believe that an increasing trend of groundwater depletion is a serious matter and quick measures are needed for sustainable groundwater usage. In the sense of sustainability, the groundwater abstraction rate should be lower than the recharge rate^52–54^. Our results show that IIT are capable to reduce groundwater utilization compared to status-quo irrigation. However, such improvements can also have negative side effects like the reduction percolation losses from fields. These apparent negative losses lead, on the one hand, to a leaching of salts from the soil^10^ and, on the other hand, also to groundwater recharge. Overall, our estimates verify that the reduction in groundwater abstraction is larger than field losses, resulting in an overall recharge of the groundwater body.

Our overall findings reveal that the status quo irrigation practices are favorable where groundwater depletion and CO_2_ emissions are not such a problem, i.e., the lower part of the Indus basin (Sindh). While IIT could be valued in areas where groundwater consumption is large (i.e., center Punjab), and where groundwater depletion rates, irrigation costs and CO_2_ emissions are high. This is somehow contradicting the current national water policy of Pakistan, as the government is trying to implement IIT throughout the whole country^55^. This is because, the national water policy is based on the country’s overall water management challenges without considering any spatiotemporal variability of the status quo irrigation practices and their economic and ecological impact. In line with our findings, we recommend that IIT should be adopted particularly in regional hotspots where the status quo irrigation practices have a strong negative environmental impact and the economic performance is particularly bad.

## Conclusions

In this paper, we assess the economic and environmental impact of status-quo irrigation settings and alternative IIT in the Indus basin of Pakistan. We evaluate four scenarios by using different energy sources for improved irrigation systems and compare the overall outcomes with the status-quo irrigation method. Results indicate that a reduction in groundwater depletion is possible for all scenarios. CO_2_ emissions can be reduced, particularly when solar energy is considered for power supply. For all other cases, the current status-quo is superior. We further show that irrigation costs increase in all scenarios compared to the status-quo. However, subsidized electricity is the preferable power source for IIT followed by solar energy, non-subsidized electricity, and diesel engines. From a cost-point view, we recommend solar energy as the second-best option for farmers if no subsidized electricity is available.

Apart from the benefits, the solar system might require a large area for panels installation, which could cause a reduction in the availability of cultivated land^56^. Nevertheless, state-of-the-art agro voltaic systems could offer a solution for the future, providing energy supply, reducing drought stress and water consumption and thereby improving water use efficiency^56,57^.

This study is conducted assuming the current boundary conditions of agricultural production in Punjab and Sindh, i.e., irrigation needs, available water and energy resources, as well as energy prices. In future studies, the impact of climate change, resulting glacier melt as well as demographic changes should be taken into account when developing sustainable irrigation practices for Pakistan. We also recommend that future estimates of irrigation costs should also include global CO_2_ market prices by considering externalities of CO_2_ emissions^58^.

Further aspects that should be picked up in future sustainability analysis are related to stakeholders and landowners. Our study does not consider any personal preferences and choices of farmers, which might result in barriers when adopting new irrigation technologies. And finally, rebound effects should also be considered when new technologies hit the market^59,60^, particularly if water costs are low and solar powered pumping becomes an economic alternative on the long-term.

## Materials and methods

### Modeling framework

**Figure 6:**
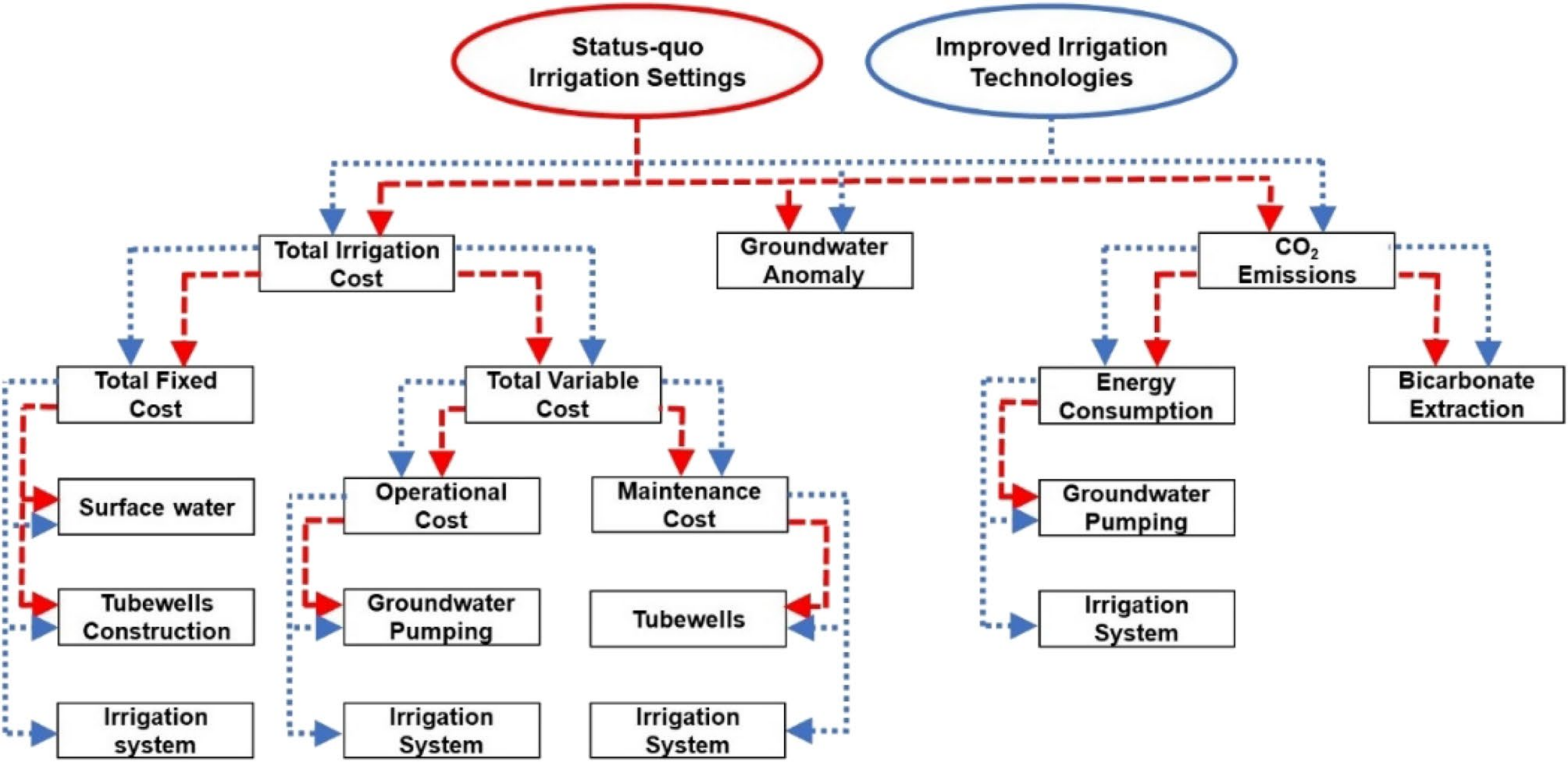
Methodological steps to estimate the economic and environmental impact of the status-quo irrigation settings and IIT.

In this study, we develop an economic-environmental-modeling framework to evaluate the economic and environmental impacts of the status-quo irrigation practices and a variety of scenarios with IIT. The model is written in python by using the SciPy package. The modeling approach uses gridded data and makes use of information such as the irrigation requirements, harvested area, crop water consumption, groundwater level, energy use required for pumping water, water prices and energy costs. The methodological steps of the modeling framework are summarized in Fig. 6, and the calculation methods are described in the below section. The input data used in this study are given in Table S1 as a supplementary material.

### Calculation methods

#### Irrigation requirements

IRR_area_ are calculated for the entire area by combining all crop’s productive (IRR_prod_) and unproductive (IRR_unprod_) consumptions of irrigation water along with the leaching requirements (LR) [Eq. (1)]. IRR_prod_ contributes to crop growth, while IRR_unprod_ covers the water losses in line with the efficiency of the irrigation system (IRR_effi_). IRR_unprod_ does not result in crop production and percolates from the root zone to the groundwater or evaporates at the soil surface. These water losses partially cover the LR^61^. The LR is an additional amount of water that is otherwise needed to leach salts from the root zone by assuming the salinity tolerance limit of each crop and the salt fraction in the irrigation water^62^.

1 $${\mathrm{IRR}}_{\mathrm{area}}=\frac{{\mathrm{IRR}}_{\mathrm{prod}}}{{\mathrm{IRR}}_{\mathrm{effi}}}+\mathrm{LR}$$

with IRR_area_, IRR_prod_, and LR given in (km^3^ year^−1^) and IRR_effi_ in percentage (%).

In this study, we use data on the site-specific IRR_prod_ and LR (2002–2016) from a recently published study^10^, where uncertainties in the input data have been quantified. The dataset holds information with a spatial resolution of 0.063° for Pakistan. Muzammil et al.^10^ used SPARE:WATER, an open-source model integrated into a geographical information system to estimate the crop water balance at the grid level^61^. SPARE:WATER follows the FAO56 guidelines to determine crop water requirements^63^ and calculates the potential LR in line with the salinity tolerance limit of crops and the salt fractions in the irrigation water. For this study, we extended the simulation period of 2002–2016 from Muzammil et al.^10^ and included the years 2017 and 2018. A detailed list of model input data and parameters required to run the model is given in Muzammil et al.^10^. The climatic data is obtained from the Pakistan Metrological Department, while information on crops is provided from the Pakistan Statistics Bureau. The efficiencies of irrigation systems are taken from the FAO dataset as 60%, 75%, and 90% for surface, sprinkler, and drip irrigation, respectively^64^.

#### Surface water and groundwater use

As surface water and groundwater are used in the Indus basin to meet the irrigation demand, we estimate the surface water share (km^3^ year^−1^) from a dataset of annual canals supply. The data is preprocessed to exclude the off-farm water losses assuming a conveyance efficiency of 70%^3,65^. The volume of groundwater abstraction (km^3^ year^−1^) is determined by subtracting the available surface water in the fields from IRR_area_.

#### Irrigation costs

TC_area_ (million US$ year^−1^) are estimated by adding the TFC_area_ and TVC_area_ [Eq. (2)]:

2 $${\mathrm{TC}}_{\mathrm{area}}={\mathrm{TFC}}_{\mathrm{area}}+{\mathrm{TVC}}_{\mathrm{area}}$$

##### TFC_area_

TFC_area_ are estimated by adding its components on a regional basis [Eq. (3)], i.e., SWP_area_, TCC_area_, and irrigation system costs (ISC_area_).

3 $${\mathrm{TFC}}_{\mathrm{area}}={\mathrm{SWP}}_{\mathrm{area}}+{\mathrm{TCC}}_{\mathrm{area}}+{\mathrm{ISC}}_{\mathrm{area}}$$

where SWP_area_ (million US$ year^−1^) results from summing up the products of costs occurring for surface water for crop irrigation (US$ ha^−1^) times their harvested area (ha year^−1^). TCC_area_ (million US$ year^−1^) is estimated by dividing the initial costs of all tubewells (million US$) for a given area from their average lifetimes (years). The initial costs of tubewells are projected by combining the construction costs of all diesel and electric operated tubewells. The TCC_area_ vary and depend on groundwater level and power required for pumping groundwater^1^. ISC_area_ are calculated by summing up the product of all crops’ irrigation system costs per hectare (US$ ha^−1^) times their harvested area (ha year^−1^). Note that the annual ISC_area_ are split in halves for the crops of the two growing seasons Kharif and Rabi, respectively. ISC_area_ are derived from dividing the initial costs of the systems by their average lifetimes (years). The status-quo irrigation system is based on gravity, therefore ISC_area_ for surface irrigation are negligible^27^. The initial costs of the improved irrigation system vary from crop to crop and by changing the power source.

##### TFC_area_

We use Eq. (4) to calculate the regional value of TVC_area_ by adding its components, i.e., the operational costs (OC_area_) and MC_area_:

4 $${\mathrm{TVC}}_{\mathrm{area}}={{\mathrm{OC}}_{\mathrm{area}}+\mathrm{MC}}_{\mathrm{area}}$$

We further divide OC_area_ into two parts, i.e., the groundwater pumping costs (GPC_area_) and the operational costs of the irrigation system (OCS_area_). Accordingly, MC_area_ are composed of the maintenance costs of the tubewells (MCT_area_), and the maintenance costs of the irrigation system (MCS_area_).

The GPC_area_ (million US$ year^−1^) is based on the costs for the energy sources diesel and electricity. The share of diesel and electric pumping in the study area is estimated by using the fraction of diesel and electric operated tubewells in a grid cell. GPC_area_ are projected by adding the groundwater pumping costs of diesel (GPC_area(d)_) and electric (GPC_area(e)_) operated tubewells. Both, GPC_area(d)_ and GPC_area(e)_, are calculated by summing up the product of the tubewell abstracted groundwater volumes (m^3^) times the pumping costs (US$ m^−3^). Pumping costs are calculated by multiplying the energy consumed (kWh) per m^3^ pumped groundwater and the energy price (US$ kWh^−1^). The energy consumption is determined from Eq. (5) where V, TDH, and η_pp_ are abstracted groundwater volume (m^3^), total dynamic head (m), and pumping plant efficiency (%), respectively^66^. In this study, the energy price for the electric source is used directly as the given electricity price in the country (US$ kWh^−1^) while for diesel consumption, fuel price (US$ L^−1^) is converted into an energy price (US$ kWh^−1^) by multiplying fuel price with a conversion factor of 0.11^66^.

5 $$\mathrm{Energy }(\mathrm{kWh})= \frac{\mathrm{ V}\times \mathrm{TDH }}{ 367 \times {\upeta }_{\mathrm{pp}}}$$

The OCS_area_ (million US$ year^−1^) consists of the energy and labor costs of the irrigation system. The energy costs for the surface irrigation method are negligible as its operation is based on gravity^67^. For the pressurized irrigation system, energy demand is estimated by multiplying the energy required to run the irrigation system (kWh year^−1^) and the energy price (US$ kWh^−1^), being either diesel or electricity. The energy consumption is estimated from Eq. (5) where TDH indicates the total head required to run the irrigation system, i.e., the operational head, friction losses, and suction lift. Labor costs are calculated by summing up the product of labor charges (US$ ha^−1^) and the harvested area (ha year^−1^).

MCT_area_ (million US$ year^−1^) is calculated by summing up the annual maintenance costs of diesel and electric operated tubewells in the region. The maintenance costs of diesel and electric operated tubewells are estimated by multiplying the maintenance costs per tubewell and the number of electric and diesel operated tubewells in the study area.

Finally, the MCS_area_ (million US$ year^−1^) contains repair and cleaning costs of the watercourses, which is calculated by multiplying the maintenance costs (US$ ha^−1^) and the total harvested area (ha year^−1^). For IIT, maintenance costs cover repair and security costs of the system. We estimate it as 5% of the total operational costs^68^.

#### Groundwater storage

The annual aquifer recharge (mm) is estimated from the Water Table Fluctuation method by adding the groundwater storage anomaly (mm) and the depth of pumped groundwater from the aquifer (mm)^69,70^. We use monthly terrestrial water storage data from the Gravity Recovery and Climate Experiment (GRACE) to estimate the groundwater storage anomaly. GRACE data has been validated for Pakistan in past studies^50,71^. In this study, we apply the GRACE Mascon solution, which does not need post-processing filtering and which is less depending on scale factors^72^. Groundwater storage anomaly is derived by subtracting the surface water storage (soil moisture, canopy water, snow water) from the terrestrial water storage. The surface water storage is estimated up to 2 m of the soil column from the land surface model (NOAH) dataset of the GLDAS product, which has been used in several regions where in situ measurements are not available^73–76^.

Further, we calculate the contributions of the fields’ percolation losses to total recharge. For the status-quo irrigation settings, it is estimated from published data^77^. This data is simulated via the GLEAMS hydrological model, which is used at the field scale to estimate the movement of water content through percolation and contribution of recharge to the groundwater^78^. Accordingly, water percolates from fields to the groundwater storage in the Indus basin of Pakistan at a rate of 0.314 mm day^−1^. It is assumed that this percolation is negligible for IIT where irrigation surplus is marginal^79^.

#### Carbon dioxide emissions

We estimate CO_2_ emissions from the status-quo irrigation practices and IIT, where energy consumption and bicarbonate extraction from the groundwater are considered as the major emissions sources.

##### CO_2_ emissions from energy consumption

There are two energy consumption sources related to irrigation in the study area, i.e., groundwater pumping and irrigation system operation. CO_2_ emissions are calculated by following the GHG protocols scope 1 (emission sources own or controlled by individual or company, i.e., fossil fuel consumption) and scope 2 (emissions from purchased electricity)^80^. The annual mass of CO_2_ emissions depends on the amount of energy consumed (kWh year^−1^) and the types of these energy sources^81^, represented by their respective emission factors. We apply a fixed emission factor for diesel engines of 0.32021 kg CO_2_ kWh^−1^ ^82^. For electricity, we calculate with a constant value of 0.47337 kg CO_2_ kWh^−1^ based on information on the major energy sources for power production in Pakistan^83^. Note that the status − quo irrigation system is based on gravity, therefore, no CO_2_ is emitted.

##### CO_2_ emissions from bicarbonates extraction

In this study, we assume that the CO_2_ concentrations in recharging groundwater and pumped groundwater are the same. If groundwater recharge is equal to the abstraction, there are no CO_2_ emissions^7^. Hitherto, CO_2_ is emitted if groundwater is depleted and CO_2_ is sequestered in the aquifer in cases of rising groundwater levels. We estimate CO_2_ emissions/sequestration (million t CO_2_ year^−1^) by multiplying CO_2_ concentrations in the groundwater (mg L^−1^) and groundwater depletion/increase (m^3^). Groundwater depletion/increase is estimated by multiplying the groundwater storage anomaly (m) and surface area of the plain (m^2^).

The CO_2_ concentrations in the groundwater depend on atmospheric CO_2_ dissolved in water, which enters the groundwater body via percolation and thus depends on the groundwater recharge rate. During solution, CO_2_ and H_2_O split into hydrogen (H^+^) and bicarbonate (HCO_3_^−^) ions [Eq. (6)].

6 $${\mathrm{CO}}_{2}+{\mathrm{H}}_{2}\mathrm{O }\to {\mathrm{H}}^{+}+{\mathrm{HCO}}_{3}^{-}$$

It is assumed that half of the mass of total bicarbonates present in the groundwater originates from this separation. While another half is formed when the CaCO_3_ rich rock in the aquifer reacts with hydrogen ions (H^+^)^6^ [Eq. (7)]:

7 $${\mathrm{H}}^{+}+{\mathrm{CaCO}}_{3}\to {\mathrm{HCO}}_{3}^{-}+{\mathrm{Ca}}^{2+}$$

Depending on the resulting bicarbonate concentration in the groundwater, CO_2_ evolves into the atmosphere according to Eq. (8) when groundwater is pumped.

8 $${\text{Ca}}({\text{HCO}}_{3} )_{2} \to {\text{CO}}_{2} + {\text{H}}_{2} {\text{O}} + {\text{CaCO}}_{3}$$

The resulting CO_2_ concentration (mg L^−1^) in the groundwater is calculated by multiplying the molecular mass ratio of HCO_3_^−^ and CO_2_ with the bicarbonate concentration (mg L^−1^) [Eq. (9)].

9 $${\mathrm{CO}}_{2}\mathrm{ Concentration }=\frac{1}{2}\mathrm{ HC}{\mathrm{O}}_{3}^{-}\times \frac{44}{61}$$

#### Scenario development

We develop four future scenarios (SC-1 to SC-4) to derive a potential optimum plan for irrigation that reduces the irrigation costs, groundwater depletion, and CO_2_ emissions in the Indus basin. Scenarios are established by changing the status-quo irrigation methods (gravity-fed surface irrigation) to IIT as this has been identified as a preferable solution to reduce total amount of irrigation water^10^. The year 2018 is considered as a baseline to which scenarios are compared. We keep the harvested area from the baseline in the scenarios and convert surface irrigation to drip irrigation for row crops and to sprinkler irrigation for field crops. The scenarios are classified according to the energy sources required to operate the revised irrigation system. In SC-1, the diesel engines are used to operate the irrigation system, SC-2 is run on electricity but assumes subsidized prices as status quo conditions, SC-3 is also based on electricity, but considers the actual energy price, and SC-4 is defined by using solar energy.

## Supplementary Information

Supplementary Information.

## Data Availability

The required data is obtained from different departments and online sources. A list of all input datasets along with data sources is given in Table S1 as supplementary material.
