# Supplementary material for: Economic and environmental impact assessment of sustainable future irrigation practices in the Indus Basin of Pakistan
Source: Sci Rep. 2021 Dec 6;11:23466. doi: 10.1038/s41598-021-02913-9 (PMC8648764; doi:10.1038/s41598-021-02913-9)
Supplement: Supplementary file 1 — Supplementary Information. [file 41598_2021_2913_MOESM1_ESM.docx]

**Economic and Environmental Impact Assessment of Sustainable Future Irrigation Practices in the Indus Basin of Pakistan**

**Muhammad Muzammil**^1,2*^**, Azlan Zahid**^3^**, Lutz Breuer**^1,4^
^1^Institute for Landscape Ecology and Resources Management (ILR), Research Centre for BioSystems, Land Use and Nutrition (IFZ), Justus Liebig University Giessen, Giessen, 35392, Germany

^2^Department of Irrigation and Drainage, University of Agriculture, Faisalabad 38040, Pakistan

^3^Texas A&M AgriLife Research, Texas A&M University System, Dallas, TX 75252, United States

^4^Centre for International Development and Environmental Research (ZEU), Justus Liebig University Giessen, Giessen, 35390, Germany

# **Supplementary material**

**
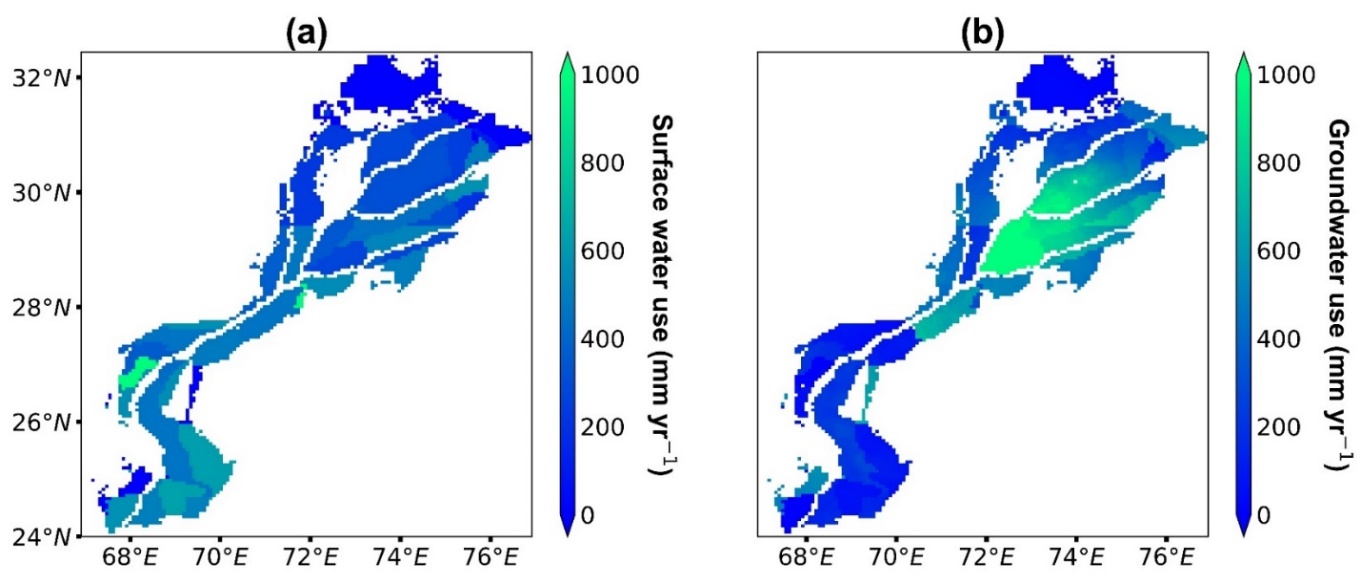
**

**Figure S1.** (**a**) Surface water and (**b**) groundwater share in irrigation water consumption.

**Table S1.** Model input data.

| **Dataset** | **Units** | **Resolution** | **Data Sources** |
| --- | --- | --- | --- |
| **Surface Irrigation** |  |  |  |
| Water price | US$ ha^−1^ | Spatial dataset | Pakistan Statistic Bureau (https://www.pbs.gov.pk/publications) |
| Labor and maintenance cost | US$ ha^−1^ | − | Directorate of Agriculture (Economics and Marketing)  (http://www.amis.pk/Surveys.aspx) |
| **Improved Irrigation** |  |  |  |
| System cost | US$ ha^−1^ | − | Punjab Irrigated Agriculture Productivity Improvement Project (PIPIP) |
| Pumping plant efficiency | % | − | Punjab Irrigated Agriculture Productivity Improvement Project (PIPIP) |
| Average lifetime | Years | − | Razzaq et al.^1^ |
| Labor cost | US$ ha^−1^ | − | Mian et al.^2^ |
| Maintenance cost | US$ ha^−1^ | − | Buchanan^3^ |
| **Tubewells** |  |  |  |
| Density | diesel and electric fraction | Spatial dataset | Pakistan Statistic Bureau (https://www.pbs.gov.pk/publications) |
| Initial cost | US$ | − | Qureshi et al.^4^ |
| Average life | Years | − | Johnson^5^ |
| Pumping plant efficiency | % | − | Pervaiz^6^ |
| Groundwater lift | m | Point dataset | Irrigation and Power Department |
| Maintenance cost | US$ | − | Qureshi et al.^7^ |
| **CO_2_ Emission** |  |  |  |
| Groundwater depletion | mm yr^−1^ | Spatial dataset | GRACE (https://www.gfz-potsdam.de) |
| HCO_3_ concentration | Mg L^−1^ | Point dataset | Pakistan Council in Research in Water Resources  (http://pcrwr.gov.pk/water-quality-reports) |
| Emission factor | − | − | Brander et al.^8^, Wang et al.^9^ |
| **Miscellaneous** |  |  |  |
| Location of irrigated areas |  |  | IWMI^10^ |
| Conversion rate Pakistani rupee to US$ | − | − | Pakistan Statistic Bureau (https://www.pbs.gov.pk/publications) |
| Electricity price | US$ kWh^−1^ | − | Pakistan Statistic Bureau (https://www.pbs.gov.pk/publications) |
| Diesel price | US$ L^−1^ | − | Pakistan Statistic Bureau (https://www.pbs.gov.pk/publications) |

**Table S2.** Scenarios analysis of IIT.

|  |  | **Irrigation Costs**  (million US$) | | | | | | | |  | **Groundwater anomaly** (mm) |  | **CO_2_ emission**  (million tons) | | | |  |
| --- | --- | --- | --- | --- | --- | --- | --- | --- | --- | --- | --- | --- | --- | --- | --- | --- | --- |
|  | Fixed | | |  | Variable | | |  | Total |  |  |  | Energy consumption | Bicarbonates extraction |  | Total | |
|  | Surface water | Tubewells | Irrigation system |  | Operational | | Maintenance |  |  |  |  |  |  |  |  |  |  |
|  |  |  |  |  | GW pumping | System operation |  |  |  |  |  |  |  |  |  |  |  |
| Acronym | SWP_area_ | TCC_area_ | ISC_area_ |  | GWP_area_ | OCS_area_ | MC_area_ |  | TC_area_ |  |  |  |  |  |  |  | |
| Baseline | 31 | 61 | 0 |  | 855 | 0 | 624 |  | 1,571 |  | −780 |  | 3.7 | 1.6 |  | 5.3 | |
| SC−1 | 31 | 61 | 1,302 |  | 350 | 2,134 | 312 |  | 4,190 |  | 280 |  | 27.5 | −0.5 |  | 27 | |
| SC−2 | 31 | 61 | 1,330 |  | 350 | 560 | 234 |  | 2,566 |  | 280 |  | 14.5 | −0.5 |  | 14 | |
| SC−3 | 31 | 61 | 1,330 |  | 350 | 1,534 | 282 |  | 3,588 |  | 280 |  | 14.5 | −0.5 |  | 14 | |
| SC−4 | 31 | 61 | 1,850 |  | 350 | 0 | 505 |  | 2,797 |  | 280 |  | 1.5 | −0.5 |  | 1 | |

1. Razzaq, A. *et al.* An Economic Analysis of High Efficiency Irrigation Systems in Punjab, Pakistan. *Sarhad Journal of Agriculture* 9 (2018).

2. Mian, M. A., Lukeová, D. & Krepl, V. Farmer’s Perception Regarding Effectiveness of Drip Irrigation System in Attock, Pakistan. in 1 (2019).

3. Buchanan, J. R. *PB1721-Irrigation Cost Analysis Handbook*. (The University of Tennessee Agricultural Extension Service, 2002).

4. Qureshi, A. S., Gill, M. A. & Sarwar, A. Sustainable groundwater management in Pakistan: challenges and opportunities. *Irrigation and Drainage* **59**, 107–116 (2010).

5. Johnson, S. H. Large-Scale Irrigation and Drainage Schemes in Pakistan: A Study of Rigidities in Public Decision Making. *Food Research Institute Studies* **18**, 1–32 (1982).

6. Pervaiz, S. Groundwater Management Using Vertical Electrical Sounding Survey and Tubewell Auditing at Farmers’ Fields. (University of Agriculture, 2010).

7. Qureshi, A. S., Shah, T. & Akhtar, M. *The groundwater economy of Pakistan*. (International Water Management Institute, 2003).

8. Brander, M., Sood, A., Wylie, C., Haughton, A. & Lovell, J. Technical Paper | Electricity-specific emission factors for grid electricity. 22 (2011).

9. Wang, J. *et al.* China’s water–energy nexus: greenhouse-gas emissions from groundwater use for agriculture. *Environmental Research Letters* **7**, 014035 (2012).

10. IWMI. Irrigated area mapping: Asia and Africa :: IWMI Data & Tools. *International Water Management Institute (IWMI)* http://www.iwmi.cgiar.org/2018/06/irrigated-area-mapping-asia-and-africa/ (2018).
